# Asymmetries and relationships between muscle strength, proprioception, biomechanics, and postural stability in patients with unilateral knee osteoarthritis

**DOI:** 10.3389/fbioe.2022.922832

**Published:** 2022-09-16

**Authors:** Ziquan Zeng, Jiaxin Shan, Yilong Zhang, Yi Wang, Congcong Li, Junyi Li, Weijian Chen, Zixuan Ye, Xiangling Ye, Zehua Chen, Zugui Wu, Chuanxi Zhao, Xuemeng Xu

**Affiliations:** ^1^ The Fifth Clinical College of Guangzhou University of Chinese Medicine, Guangzhou, China; ^2^ Guangdong Provincial Second Hospital of Traditional Chinese Medicine, Guangzhou, China

**Keywords:** knee osteoarthritis, muscle strength, proprioception, biomechanics, postural stability

## Abstract

**Background:** The pathological mechanism of knee osteoarthritis (KOA) is unknown. KOA degeneration may be associated with changes in muscle strength, proprioception, biomechanics, and postural stability.

**Objective:** This study aimed to assess asymmetries in muscle strength, proprioception, biomechanics, and postural stability of bilateral lower limbs in patients with unilateral KOA and healthy controls and analyze correlations between KOA and these parameters.

**Methods:** A total of 50 patients with unilateral KOA (age range: 50-70) and 50 healthy subjects were recruited as study participants (age range: 50-70). Muscle strength, proprioception, femorotibial angle (FTA), femoral condylar–tibial plateau angle (FCTP), average trajectory error (ATE), and center of pressure (COP) sways areas were accessed in study participants, and the correlation between these variables was investigated.

**Results:** In patients with unilateral KOA, lower limb muscle strength was significantly lower on the symptomatic side than on the asymptomatic side (*p* < 0.01), while the proprioception (degree error), FTA, FCTP, and ATE were substantially higher compared to the asymptomatic side (*p* < 0.01). However, no significant difference was observed in the healthy controls (*p* > 0.05). Patients with unilateral KOA had lower muscle strength than healthy controls (*p* < 0.05), but their proprioception (degree error: the difference between the target and reproduction angles), ATE, and COP sway areas were higher (*p* < 0.05). Muscle strength was found to be negatively correlated with ATE and COP sways areas (*p* < 0.05), whereas proprioception (degree error) was positively correlated with ATE and COP sways areas (*p* < 0.05) in all study participants. However, no correlation was found between FTA, FCTP, and ATE, COP sways areas in patients with unilateral KOA (*p* > 0.05).

**Conclusion:** In patients with unilateral KOA, muscle strength, proprioception, biomechanics, and postural stability of bilateral limbs are asymmetrical in unilateral KOA patients. Muscle strength, proprioception, and postural stability are significantly associated variables, and changes in these variables should be considered in KOA prevention and rehabilitation.

## Introduction

Knee osteoarthritis (KOA) is a chronic disease characterized by pain, deformity, and joint dysfunction, negatively affecting a patient’s quality of life (Hunter David and Bierma-Zeinstra Sita, 2019; Katz Jeffrey et al., 2021). Although some studies have explained the pathophysiological mechanisms of KOA, the causes of KOA are mixed, and it is important to thoroughly investigate and comprehend the modifiable potential risk factors for KOA.

Other studies have found that postural stability is an important factor in KOA (Tetsuo. et al., 2006; Hsieh et al., 2013; Petrella et al., 2017). Postural stability is a complex process that involves sensory nerve transmission, motor control, and central integration for its maintenance (Yoshikawa et al., 2008; Ryan Edwin et al., 2010). Patients’ basic ability to live and health-related quality of life suffer as their postural stability deteriorates (Swanik et al., 2004; Leveille Suzanne et al., 2009). Furthermore, decreased postural stability can result in decreased physical function, an increased risk of falling, accelerated progression of KOA, and possibly even more severe consequences in patients with KOA (Tinetti et al., 1995; Robinson et al., 2002). It is critical to assess postural stability in KOA patients and investigate the factors that influence postural stability. Reduced postural stability in KOA patients, on the other hand, is a multifactorial problem involving multiple components such as muscle atrophy, neuromuscular coordination, altered muscle, nerve activation, and so on (Hassan et al., 2001; Hinman et al., 2002; Tetsuo. et al., 2006; Hirata et al., 2019).

The quadriceps are among the most important muscles for maintaining knee joint stability of the knee joint and a prerequisite for maintaining normal postural stability in KOA patients (Horlings Corinne et al., 2008; Petrella et al., 2017). It was found previously that KOA patients have lower quadriceps strength associated with other factors such as pain, joint loading, joint structural damage, and knee function (McAlindon et al., 1993; Guccione et al., 1994). Although a link has been established between decreased muscle strength and self-reported knee instability (Knoop et al., 2012), the relationship between reduced muscle strength and postural stability remains inconclusive. In addition, several studies have found that muscle strength, proprioception, pain, and range of motion are factors that contribute to decreased balance in patients with KOA and should be considered when evaluating postural balance (Mohammadi et al., 2008; Sanchez-Ramirez et al., 2013; Takacs et al., 2015; Liu et al., 2017). At the same time, improving muscle strength can improve patients’ quality of life and reduce the risk of falls (Gezginaslan et al., 2018). However, some research has found that increasing muscle strength does not affect KOA (Sharma and Dunlop Dorothy, 2003). In addition, several systematic reviews and clinical studies have shown that decreased muscle strength is associated with the development of KOA (Hootman et al., 2004; Omori et al., 2013; Øiestad et al., 2015). Although muscle strength can predict the development of KOA, this relationship may be influenced by other factors (Culvenor Adam et al., 2017). There is still some uncertainty about the relationship between muscle strength and KOA. As muscle strength is a modifiable factor, it is critical to understand the effect of changes in muscle strength on postural stability in KOA patients.

Proprioception is the perception of position or movement of a limb or joint in space (Sharma, 1999). The proprioception of knee joint is primarily dependent on the input of mechanoreceptors present in muscles, ligaments, joint capsule, and meniscus (Bennell Kim et al., 2003). It plays a vital role in maintaining knee stability and coordinating neuromuscular control (Fischer-Rasmussen and Jensen, 2000) that helps proper knee function and reduces knee injury risk during abnormal movements or postures (Felson David et al., 2009; Knoop et al., 2011). Proprioception impairment can result in abnormal knee movements and decreased neuromuscular coordination (Fischer-Rasmussen and Jensen, 2000). However, some studies suggest that there may be no association between impaired proprioception and postural swaying (Hassan et al., 2001). There is inadequate clinical evidence suggesting whether bilateral knee proprioception is altered in unilateral KOA patients and whether the proprioception and postural stability are related. Investigating the changes in proprioception and their relationship with postural stability is significant for reducing the adverse effects of postural instability on KOA.

Femorotibial angle (FTA) and femoral condylar–tibial plateau angle (FCTP) are important biomechanical reference factors for evaluating varus or valgus and joint space narrowing in KOA (Nakagawa et al., 2015; Higano et al., 2016). Studies also illustrate the link between varus deformity of the knee and progression of medial KOA (Sharma et al., 2001). The biomechanical changes in lower limbs are an important factor in KOA. However, they do not predict the onset of KOA, and the association between these biomechanical changes and KOA remains debatable (Hunter David et al., 2007). Although the valgus or valgus angle of the knee joint in KOA patients may predict lower limb balance function (Hunt et al., 2010). Still, the relationship between biomechanical factors like FTA, FCTP, and postural stability remains unclear.

Previous studies have reported changes in muscle strength, proprioception, biomechanics, and postural stability in patients with KOA (Hassan et al., 2001; Hassan et al., 2002; Hall et al., 2006; van der Esch et al., 2007; Mohammadi et al., 2008; Sanchez-Ramirez et al., 2013; Vårbakken et al., 2019; Domínguez-Navarro et al., 2020). However, the differences between the symptomatic and asymptomatic sides of patients with unilateral KOA remain an interesting topic to explore. This study aimed to evaluate whether muscle strength, proprioception, biomechanics, and postural stability of bilateral lower limbs were symmetrical in patients with unilateral KOA and healthy controls and analyze the correlation among these variables. This study may contribute to a better understanding of the pathophysiological mechanism of KOA and serve as a reference for KOA prevention and rehabilitation.

## Methods

### Study participants

The study included 50 patients with unilateral KOA and 50 healthy individuals of the same age and gender. All patients with unilateral KOA were from the Department of Orthopedics, Guangdong Second Traditional Chinese Medicine Hospital. Inclusion criteria for KOA patients included the following: 1) The age range is 50–70 years old, 2) Kellgren/Lawrence (K/L) grade range from 1 to 3 (Kellgren and Lawrence, 1957), 3) VAS scores range from 1 to 6, 4)BMI≤30 kg/m^2^, 5) met the diagnostic criteria of the American College of Rheumatology (Altman et al., 1986) and had symptoms in the unilateral knee joint, and 6) there is primarily OA in the medial compartment of the knee joint. Exclusion criteria for KOA patients included the following: 1) congenital or traumatic lower limb deformity, 2) presence of neurological disorders such as Parkinson’s disease, spinal-related disorders, vertigo or stroke, 3) the presence of hip, ankle, or waist disease, 4) the knee joint has received systematic treatment in the last 6 months, 5) the knee joint or other parts of the lower limbs have a history of surgery, 6) lower limb fracture or other trauma histories, 7) OA of the lateral compartment of knee joint, and 8) other inflammatory arthritis.

A total of 50 healthy volunteers made up the control group. The participants were selected from patients’ families at Guangdong Second Traditional Chinese Medicine Hospital and the surrounding communities. Inclusion criteria included the following: 1) The age range is 50–70 years old, 2) no lower limbs trauma or surgical history, 3) no diseases of the knee, ankle, or hip joint, 4) no lower limbs deformity and no musculoskeletal diseases in other parts of the lower limbs, 5) no musculoskeletal diseases in the waist, and 6) no neurological diseases.

The research team recruited and screened participants who met the inclusion criteria. They collect all participants’ demographic and clinical information, including their name, gender, age, height, weight, disease duration, pain location, etc. Two different groups of researchers were deployed to collect the demographic and clinical information and information about muscle strength, proprioception, biomechanics, and postural stability to eliminate the risk of biases.

### Measurement of quadriceps strength

The Biodex™ (Shirley, NY, United States) isokinetic dynamometer system (Biodex System 3 Pro) was used to measure the muscle strength of the knee extensor muscle (quadriceps femoris). This device is reliable and accurate for measuring muscle strength (Drouin Joshua et al., 2004). Participants sat with their hands folded in front of their chests, hips flexed to 90°, and waists restrained with system-equipped straps. The axis of rotation of the knee joint was kept aligned with the axis of rotation of the isokinetic dynamometer system by placing an immobilization pad over the ankle joint. The measured angular velocity was set to 60°/s (Chang et al., 2019). Each participant took two pretest values before the start of muscle strength measurements. At the beginning of the analysis, the participants were directed to repeat the flexion and extension of the knee joint five times continuously at a speed of 60°/s, using their maximum muscle strength. Muscle strength was represented by normalized mean peak torque (Nm) divided by body weight (Kg), with higher values indicating greater muscle strength. The entire measurement process was conducted in a standardized manner for all participants under the same directions and encouragement. The results were collected in a duplicate manner and presented as mean values.

### Measurement of knee proprioception (degree error)

The Biodex™ (Shirley, NY, United States) isokinetic dynamometer system (Biodex System 3 Pro) was used to assess the knee proprioception. Proprioception was measured as knee joint position sense by applying the passive extension-active reproduction method by selecting the appropriate measurement program provided by the system (van der Esch et al., 2007). This knee proprioceptive measurement method is widely used, valid, and reproducible (van der Esch et al., 2007). Participants were asked to keep their eyes closed (visually shielded), maintain the same position as the muscle strength measurement, and hold a device with a button during the analysis. Two pre-measurements were performed for each participant before the actual analysis. 60° was set as a target angle at the beginning of the measurement. The calf at 90° of knee flexion is considered a starting point that passively expands to 60° (target angle) at a rate of 10°/s. After holding the target angle position for 10 s, the lower leg was returned to the starting position (90° of knee flexion). The participants were then instructed to actively extend the lower leg to reproduce the target angle (60°). When the participant believes that they have reached the target angle, a button on the handheld device is pressed. The system will complete the experiment and record the angle at that point (reproduced angle). The proprioceptive measurement program automatically computes the difference between the target and reproduction angles. This difference reflects the participant’s perception of the knee joint. The smaller value of difference indicates a better proprioceptive sense (van der Esch et al., 2007). The entire measurement process was carried out in a standardized manner for all participants under the same directions and encouragement. Results were collected in a duplicate manner and presented as mean values.

### Measurement of the biomechanics (FTA and FCTP) of the lower limbs

**FIGURE 1:**
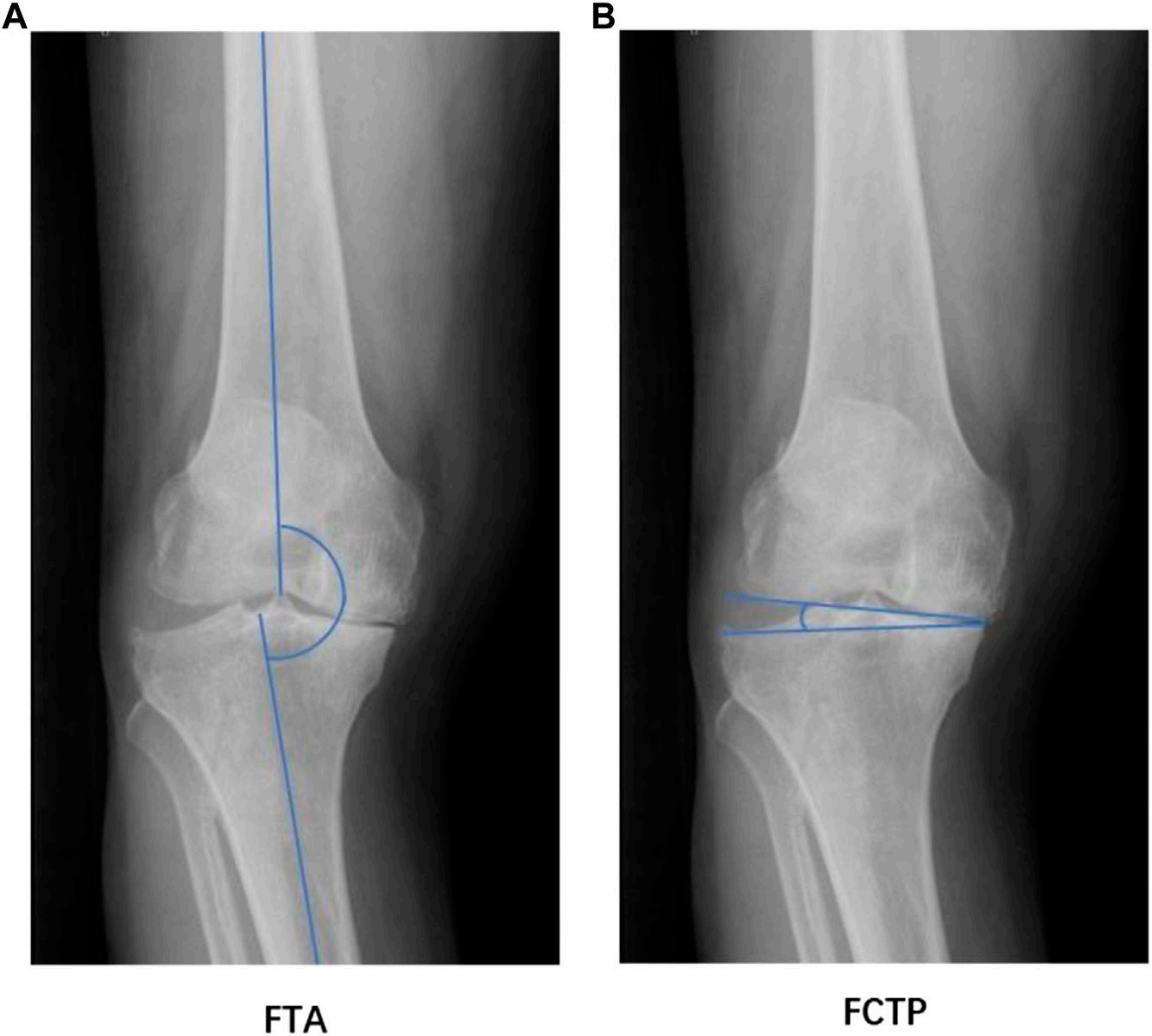
FTA **(A)** and FCTP **(B)**.

Digital radiography was used to measure the femoral-tibial angle (FTA) and the femoral condyle-tibial plateau angle (FCTP) in KOA patients. FTA and FCTP were obtained on standard weight-bearing anteroposterior radiographs. The measurements were performed by an orthopedic surgeon with 10 years of relevant experience. FTA is the angle between the femoral and tibial anatomical axis (Nakagawa et al., 2015). Concurrently, FCTP is the angle between the tangents to the subchondral plates of the femoral and tibial condyles (Nakagawa et al., 2015). FTA and FCTP are important parameters for evaluating the biomechanics of lower limbs because they can indicate the varus or valgus deformity of the knee joint (Nakagawa et al., 2015; Higano et al., 2016). FTA and FCTP are depicted in Figure 1.

### Measurement of Postural Stability (ATE and COP sways areas)

A Pro-kin 254P dynamic and static balancer (TecnoBody, Dalmine, Italy) were used to measure dynamic stability (average trajectory error, ATE) and static stability (COP sways area). Previously reported data has confirmed the reproducibility and validity of the device for measuring postural stability (Chen et al., 2020). Two pre-measurements were performed for each participant before the actual analysis. During the ATE measurement, the participant stood on the operating board of the dynamic and static balancer. The operating board of the instrument was controlled by one lower limb as directed by the system. Five circles were drawn along the system specified trajectory within the specified time (120 s). The left and right limbs illustrate the system-provided trajectory in anticlockwise and clockwise directions. During the analysis, the participants are encouraged to use the fastest speed and highest accuracy to describe the system trajectory. Following the procedure, the system will automatically record ATE. The smaller value of ATE suggests better dynamic stability.

**FIGURE 2:**
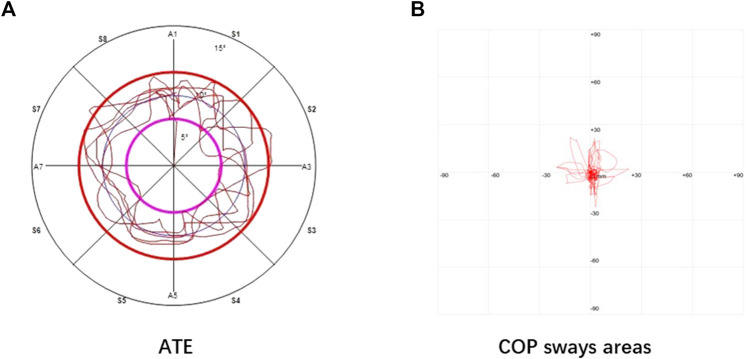
ATE **(A)** and COP sway areas **(B)**.

Similar to ATE measurement, during COP sways area analysis, the participant stood on the operating board of the instrument with a lower. Following the dynamic stability measurement procedure, the participants stand statically for 30 s on both lower limbs, with both upper limbs naturally drooping on the sides of the body. During measurement, the patients were encouraged to stand as firmly as possible on the operating board of the dynamic and static balancing instrument. The system will automatically record COP sways areas that indicate the static stability (SA, mm^2^). The smaller value of COP sways areas represent better static stability. All readings were taken in a duplicate manner and represented as mean values. The entire measurement process was carried out in a standardized way for all participants under the same directions and encouragement. ATE and COP sway areas measurements are depicted in Figure 2.

### Statistical analysis

All statistical analyses were performed using SPSS version 26.0 (IBM, Armonk, New York, United States). Descriptive statistics were performed on the participant’s demographics and clinical data and represented as mean ± standard deviation. The Shapiro-Wilk test was used to confirm the normal data distribution. The chi-square test was utilized to compare the categorical variables between the two groups. However, the differences in data among the test and control groups were compared by independent samples *t*-test (normal distribution) or Mann-Whitney *U* test (non-normal distribution). Paired Student’s t-test (normal distribution) or Wilcoxon test (non-normal distribution) were used to determine differences between bilateral lower limbs. The Pearson correlation test (normal distribution) or Spearman correlation test (non-normal distribution) were employed to determine the correlation between muscle strength, proprioception (degree error), FTA, FCTP, and ATE, COP sways areas. Statistical differences were considered significant at *p* < 0.05.

## Results

### Study population

Following the inclusion and exclusion criteria, 50 patients with KOA and 50 healthy participants of the same age and gender were chosen. All participants completely follow the study protocol. The information collected indicates no statistically significant differences in age, sex, height, weight, and BMI among both groups. The basic information of all participants is shown in Table 1.

**TABLE 1:** Baseline condition of subjects (*n* = 100).

Items	PG (*n* = 50)	CG (*n* = 50)	*p*-values
Age/age range (years)	60.88 ± 5.73/ (50−70)	61.28 ± 5.73/(50−70)	0.735
Gender (male/female)	19/31	20/30	1.000
Height (m)	1.61 ± 0.41	1.62 ± 0.48	0.229
Weight (Kg)	63.30 ± 4.27	62.36 ± 5.72	0.583
BMI (Kg/m^2^)	24.21 ± 1.71	23.49 ± 2.02	0.057
Duration of disease (months)	38.12 ± 35.23	—	—
Pain location (left/right)	25/25	—	—
VAS scores	3.62 ± 1.49	—	—
K/L grade	2.32 ± 0.74	—	—

Values are mean ± standard deviation or *n*; BMI, body mass index; K/L, Kellgren/Lawrence; PG, patient group; CG, control group.

### Analysis of muscle strength, proprioception (degree error), biomechanics (FTA, FCTP), and postural stability (ATE, COP, sways areas) in patient and control groups

**FIGURE 3:**
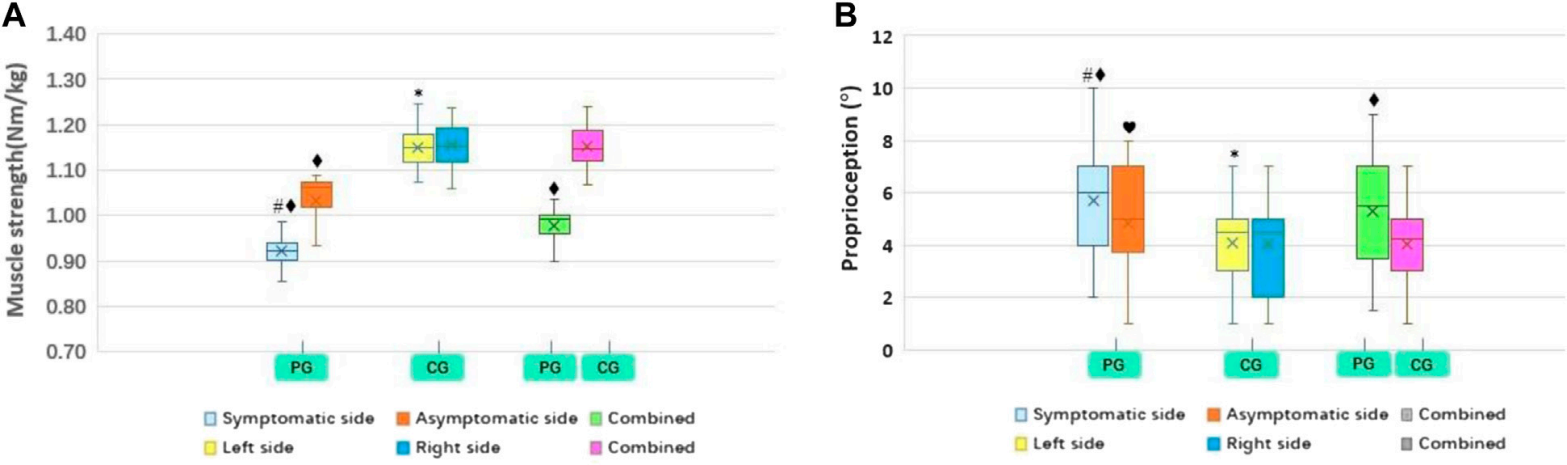
PG and CG differences in muscle strength **(A)** and proprioreception **(B)**. PG, patient group; CG, control group. Compared to contralateral, ^#^ *p* < 0.01; Compared to contralateral, *p > 0.05; Compared to CG, ^♦^ *p* < 0.01; Compared to CG, ^♥^ *p* < 0.05.

**FIGURE 4:**
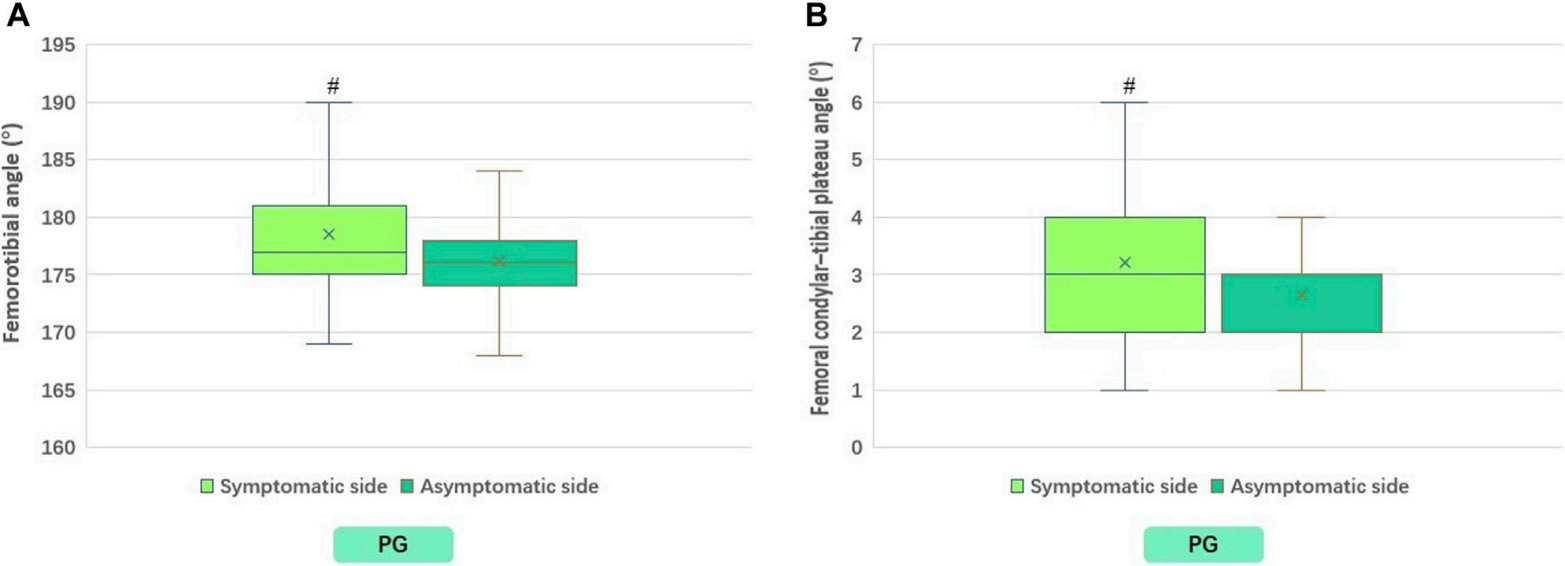
Differences between symptomatic and asymptomatic sides of PG in FTA **(A)**, and in FCTP **(B)**. PG, patient group. FTA, femorotibial angle; FCTP, femoral condylar–tibial plateau angle. ^#^ *p* < 0.01; Compared to contralateral.

**FIGURE 5:**
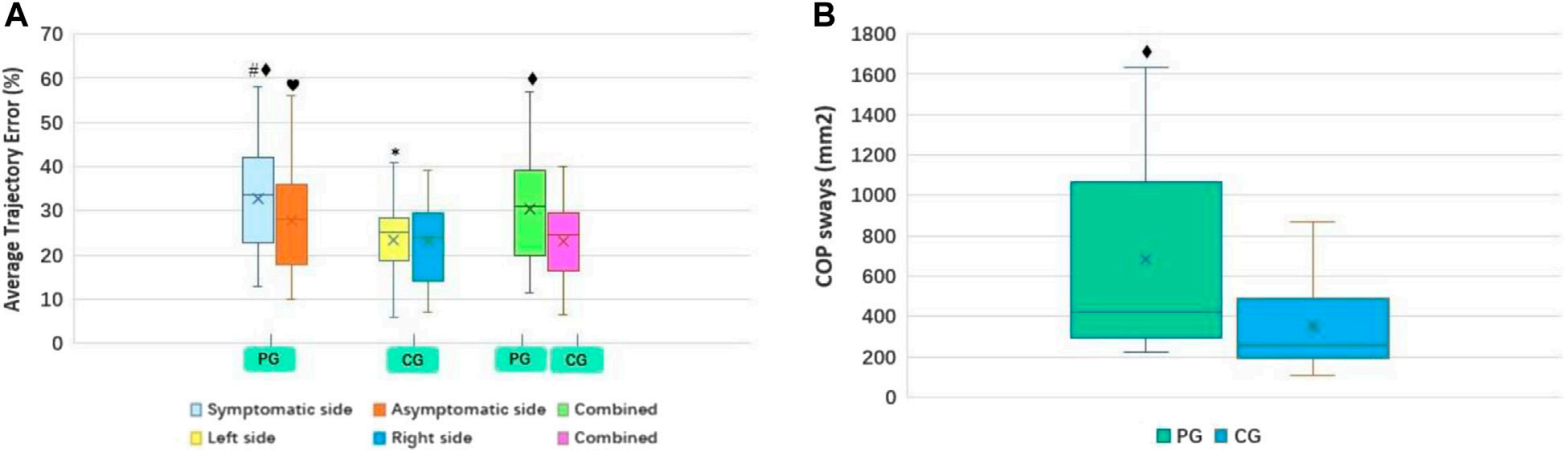
PG and CG differences in ATE **(A)** and COP sway areas **(B)**. PG, patient group; CG, control group. ATE, average trajectory error; COP, the center of pressure. Compared to contralateral, ^#^ *p* < 0.01; Compared to contralateral, *p > 0.05; Compared to CG, ^♦^ *p* < 0.01; Compared to CG, ^♥^ *p* < 0.05.

The results revealed that the lower limb muscle strength on the symptomatic side was significantly lower than that on the asymptomatic side (*p* < 0.01). Concurrently, the proprioception (degree error), FTA, FCTP, and ATE were significantly higher than those on the asymptomatic side (*p* < 0.01) (Table 2; Figures 3–5). However, there was no significant difference in the muscle strength, proprioception (degree error), and ATE of the bilateral lower limbs among the control group (*p* > 0.05) (Table 2; Figures 3, 5). The muscle strength on the symptomatic, asymptomatic and bilateral sides of the patient’s group were significantly lower than those of the control group (*p* < 0.01) (Table 2; Figure 3 **)**. The proprioception (degree error) and ATE on symptomatic, asymptomatic, and bilateral sides of the patient group were significantly higher than those of the control group (*p* < 0.01, *p* < 0.05, and *p* < 0.01, respectively) (Table 2; Figure 3). Similarly, COP sways areas in the patient group were significantly higher than that in the control group (*p* < 0.01) (Table 2; Figure 5).

**TABLE 2:** Comparison of parameters between the two groups (*n* = 100).

Parameters	PG (*n* = 50)			CG (*n* = 50)		
	Symptomatic side	Asymptomatic side	Combined	Left side	Right side	Combined
Muscle strength (Nm/kg)	0.92 ± 0.03^#♦^	1.03 ± 0.04^♦^	0.97 ± 0.03^♦^	1.15 ± 0.04*	1.15 ± 0.05	1.15 ± 0.04
Proprioception (°)	5.72 ± 2.17^#♦^	4.86 ± 1.96^♥^	5.29 ± 2.03^♦^	4.06 ± 1.63*	4.04 ± 1.57	4.05 ± 1.45
FTA (°)	178.52 ± 5.25^#^	176.14 ± 4.20	177.33 ± 4.65	—	—	—
FCTP (°)	3.22 ± 1.34^#^	2.64 ± 1.12	2.93 ± 1.19	—	—	—
ATE (%)	32.68 ± 11.62^#♦^	27.84 ± 11.56^♥^	30.26 ± 11.49^♦^	23.28 ± 9.15*	23.06 ± 9.17	23.17 ± 9.09
COP sways areas (mm^2^)	683.08 ± 470.72^♦^			348.90 ± 208.31		

Values are mean ± standard deviation; FTA, femorotibial angle; FCTP, femoral condylar–tibial plateau angle; ATE, average trajectory error; COP, the center of pressure; PG, patient group; CG, control group. Compared to contralateral, ^#^ *p* < 0.01; Compared to contralateral, *p > 0.05; Compared to CG, ^♦^ *p* < 0.01; Compared to CG, ^♥^ *p* < 0.05.

### Correlation analysis between muscle strength, proprioception (degree error), biomechanics (FTA, FCTP), VAS, and postural stability (ATE, COP sways areas).

ATE and COP sways areas have a negative correlation (*p* < 0.05) with muscle strength and a positive association (*p* < 0.05) with proprioception (degree error) and VAS in unilateral KOA patients. Similar trends of association among these variables were found in healthy controls. However, it was also identified that no significant association was present between FTA, FCTP, and ATE, COP, sways areas in patients with unilateral KOA (*p* > 0.05). All data are presented in Table 3 as follows.

**TABLE 3:** Statistical analysis results of correlation test.

Parameters		Sides	ATE		COP sways areas	
			*r*	*p-*value	*r*	*p-value*
Muscle strength	PG	Symptomatic side	−0.380	0.006^#^	−0.338	0.016*
		Asymptomatic side	−0.312	0.028*	−0.287	0.044*
		Combined	−0.377	0.007^#^	−0.348	0.013*
	CG	Left side	−0.381	0.006^#^	−0.316	0.025*
		Right side	−0.424	0.002^#^	−0.345	0.014*
		Combined	−0.405	0.004^#^	−0.343	0.015*
Proprioception (degree error)	PG	Symptomatic side	0.330	0.019*	0.297	0.036*
		Asymptomatic side	0.339	0.016*	0.328	0.020*
		Combined	0.372	0.008^#^	0.324	0.022*
	CG	Left side	0.305	0.031*	0.367	0.009^#^
		Right side	0.385	0.006^#^	0.337	0.017*
		Combined	0.356	0.011*	0.358	0.011*
FTA	PG	Symptomatic side	0.250	0.080	0.225	0.115
		Asymptomatic side	0.133	0.359	0.147	0.307
		Combined	0.199	0.166	0.183	0.204
FCTP	PG	Symptomatic side	0.133	0.356	0.150	0.298
		Asymptomatic side	0.099	0.494	0.130	0.367
		Combined	0.118	0.413	0.155	0.281
VAS	PG	-	0.302	0.033*	0.318	0.025*

^#^Indicates *p*-value < 0.01; *Indicates *p*-value < 0.05. VAS, visual analogue scale; FTA, femorotibial angle; FCTP, femoral condylar–tibial plateau angle; ATE, average trajectory error; COP, the center of pressure; PG, patient group; CG, control group.

## Discussion

Participants’ quadriceps strength, proprioception, biomechanics, and postural stability were measured in the current study. The bilateral lower limb asymmetry in patients with unilateral KOA has been reported in pain, joint load, bone mineral density, and gait. It was found that inter-limb asymmetry is associated with KOA disease progression (Shakoor et al., 2014; Iijima et al., 2020; Nishizawa et al., 2021). Previous studies illustrate the link between decreased postural stability and KOA degeneration (Tetsuo. et al., 2006). The current study’s finding was in line with the result of various previous studies and indicated that the muscle strength and proprioception were asymmetric in unilateral KOA patients due to reduced postural stability (Hassan et al., 2001; Chen et al., 2020). Furthermore, we identified the differences in postural stability of bilateral lower limbs in patients with unilateral KOA, which is consistent with the findings of other studies (Kim et al., 2018). Asymptomatic lower limb postural stability was reduced, and bilateral lower limbs asymmetry was observed, which might be associated with variation in other contributing factors of KOA degeneration. Several studies have found that patients with unilateral hip osteoarthritis have increased dynamic loading on the contralateral knee. This initial asymmetry may be related to the patient’s efforts to alleviate pain or other symptoms in the affected limb (Shakoor et al., 2011; Shakoor et al., 2014). Maintaining postural stability is a complex, integrative process requiring the coordination of the central nervous system (CNS) with the peripheral neuromuscular system (Whipple et al., 1993; Koceja et al., 1999). The process involves coordinating sensory information transmitted by vestibular organs, vision, and somatosensory organs and various complex factors such as knee proprioception, quadriceps muscle strength, and nervous system responses (Kim et al., 2018). Persistent limbs asymmetry may be related to gait adaptations caused by changes in neuromuscular factors such as muscle strength and proprioception (Shakoor et al., 2011), suggesting a decline in the postural stability in unilateral KOA patients.

Weaker muscle strength is also associated with a higher probability of reduced knee function, which is a risk factor for KOA (Segal Neil et al., 2009; Serrão Paula et al., 2012). It was found that the quadriceps strength in the lower limbs of unilateral KOA patients was asymmetric, which was supported by the findings of Dokyung et al., who indicated that the limb with lower muscle strength was more painful than another side (Kim et al., 2018). Furthermore, many studies have found that KOA patients have lower muscle strength than healthy subjects (Messier et al., 1992; Park et al., 2016). In our study, unilateral KOA patients also had lower muscle strength in the lower limbs on the asymptomatic side than healthy controls. Pain can lead to muscle atrophy and muscle immobilization, which could be one cause of decreased muscle strength (Hurley and Newham, 1993). Steidle-Kloc et al. (2019) explain that pain in the contralateral knee affected extensor strength in the other, and this change may be related to CNS depression and pain-induced changes in afferent signals (Héroux Martin and Tremblay, 2006; Steidle-Kloc et al., 2019). Furthermore, impaired contralateral muscle strength might lead to defects in voluntary activation that suggest CNS depression (Pietrosimone Brian et al., 2011; Metcalfe Andrew et al., 2012) and affect the controlling mechanism of CNS to balance muscle activation among the limbs (Steidle-Kloc et al., 2019). Hassan et al. (2001) reported that decreased muscle strength is associated with increased postural sway, hindering the muscle’s ability to maintain postural stability. Reduced muscle strength causes muscle fatigue, resulting in a weak muscle force with increased gait variability and a negatively influenced balance control mechanism (Bigland-Ritchie and Woods, 1984; Helbostad Jorunn et al., 2007). Maintaining postural stability requires comprehensive coordination of the neuromuscular system, which involves multiple parts (Hassan et al., 2001; Hinman et al., 2002; Tetsuo. et al., 2006; Hirata et al., 2019). When there is a lack of muscle strength, CNS may use some compensatory strategies to achieve postural balance and stability (Pua et al., 2011). The postural stiffness strategy, which reduces postural sway through coordinated muscle contractions throughout the body, is an important compensatory strategy that is more pronounced in patients with knee extensor weakness (Rudolph Katherine et al., 2007; Massimo. et al., 2010). However, higher synergistic contraction of muscles around the knee, on the other hand, is associated with worse knee instability (Schmitt Laura and Rudolph Katherine, 2008). Furthermore, reduced muscle strength in patients with KOA may contribute to knee instability (Martin. et al., 2012), worsening postural stability (Turcot et al., 2011). However, extensive research has been required to investigate all these possibilities.

Proprioceptive deficits and progression of KOA are closely related (Sharma et al., 1997). According to Felson David et al. (2009) knee pain is associated with decreased proprioceptive acuity. Proprioceptive defects in KOA patients might be due to the destruction or disruption of mechanoreceptors’ structure or function in the knee joint (Schultz et al., 1984; Barrett et al., 1991). Furthermore, KOA patients have fewer mechanoreceptors in their knee ligaments (Sharma, 1999). Proprioceptive deficits might be exacerbated by a reduction in mechanoreceptors and functional impairment due to structural damage to the knee joint. The current study demonstrates that proprioceptive acuity was reduced in unilateral KOA patients than in controls. It also indicates that the proprioceptive understanding of the symptomatic knee side was weaker than that of the asymptomatic side. Sharma (1999) identify that the defects in asymptomatic knee proprioception in unilateral KOA patients increase the risk of bilateral KOA development. These deficits in the asymptomatic knee might be associated with overuse of the contralateral knee and knee injury because of limb compensation. It was identified that proprioception is important in determining the knee joint position and contributes to maintaining the knee joint stability by coordinating the quadriceps, hamstrings, and other related muscles (Segal Neil et al., 2010). Therefore, any proprioceptive impairment can significantly reduce knee stability (Sanchez-Ramirez et al., 2013). The findings suggest a direct relationship between the knee and postural stability (Turcot et al., 2011). Postural stability is maintained through comprehensive neuromuscular coordination, sensory transmission, and proprioception (Fischer-Rasmussen and Jensen, 2000; Knoop et al., 2011). In addition, neuromuscular factors such as altered proprioception can cause gait abnormalities and further inter-limb asymmetry (Shakoor et al., 2014), associated with decreased postural stability. Furthermore, neuromuscular factors such as modified proprioception can cause gait abnormalities and supplement inter-limb asymmetry (Shakoor et al., 2014), associated with reduced postural stability.

The role of the biomechanics of the frontal knee plane in KOA disease progression has been a topic of interest for researchers. The current study’s findings indicate the significant differences in the biomechanical parameters (FTA and FCTP) of the bilateral lower limbs in unilateral KOA patients. However, no correlation was found between FTA, FCTP, and postural stability. Birmingham et al. (2001) illustrated the association between postural control and KOA regression, supported by the findings of Khalaj et al. that postural stability was reduced in patients with moderate and severe KOA (Nafiseh. et al., 2014). Maintaining knee and postural stability in KOA patients requires a combination of biomechanics, muscle strength, proprioception, neurotransmission, and various other factors. Although underlying pathophysiological mechanisms of KOA are complex, the current study developed a better understanding and enlightened various risk factors involved in KOA progression. However, the role of biomechanics in KOA progression and postural stability needs to be further explored in future studies.

Although previous studies have reported changes and characteristics of muscle strength, proprioception, biomechanics, and postural stability in patients with KOA. However, studies exploring the influencing factors of unilateral KOA are still few, and the conclusions have many uncertainties. First, previous studies have primarily explored differences in bilateral KOA and healthy controls. This study focused on the differences between the symptomatic and non-symptomatic sides of patients with unilateral KOA and between patients with unilateral KOA and healthy controls. Bilateral KOA is mainly concentrated in the middle and late stages of the disease, and unilateral KOA is primarily in the early stage. Exploring the disease characteristics of unilateral KOA is of great significance for preventing and treating KOA. Second, postural stability is a topic of great interest to researchers. Previous studies have mainly used scales or subjective stability tests to assess postural stability or static postural stability alone. This study used objective metrics and assessed both dynamic and static stability. A joint assessment of dynamic and static stability is more comprehensive and accurate. In addition, this study combined imaging techniques to assess biomechanical parameters (FTA, FCTP) when assessing muscle strength, proprioception, and postural stability. Biomechanical parameters and other factors are involved in the progression of KOA and may interact with each other. Although this study did not find a direct correlation between biomechanical parameters and postural stability. However, we think this may be due to a mixture of other factors or because the biomechanical parameters of these participants are concentrated in a relatively small range. It is worth further exploring the correlation between biomechanical parameters and postural stability in the future. In future research, a larger sample size of patients and control groups might help assess the specific mechanism contributing to changes in muscle strength, proprioception, biomechanics, and postural stability in KOA patients. In conclusion, this study explores the characteristics of the influencing factors of KOA, which may help to understand the pathological mechanism of KOA better.

## Limitations

There are several limitations to this cross-sectional study. First, we included KOA patients who were admitted to the hospital and may have had more severe disease symptoms than patients who did not come to the hospital. Second, although we employed the standardized measuring procedures for all participants, the results may be influenced by subjective factors such as participants’ literacy level and acting ability. Third, the age range of participants is large, and age-related changes (e.g., sarcopenia, myasthenia) may potentially influence the results. Although the age range of the participants in our study was large, we ensured that there were no differences in age between groups, which reduced the effect of age on the results of this study. Future research should narrow the age range, for example, to within 10 years. The impact of age on KOA, and the relationship between them, will be examined independently in the future. Fourth, the factors influencing muscle strength, proprioception, and postural stability are complex, including the patient’s pain level, joint mobility, physical function, habitual physical activity, and other objective variables. However, we cannot strictly control these potential factors in clinical practice. Fifth, because medial osteoarthritis is more common, we limited our study to patients with medial KOA, which may have resulted in bias. However, disease-related changes due to varus or valgus knees may differ, and including only one type of KOA can help to reduce the impact of confounding factors. Despite some difficulties, future research should strictly control other influencing factors and further explore new findings to improve KOA management.

## Conclusion

The current study confirmed that the muscle strength, proprioception, biomechanics, and postural stability of the bilateral lower limbs of unilateral KOA patients were asymmetric. Unilateral KOA patients had lower muscle strength, proprioceptive acuity, and postural stability than healthy controls. Furthermore, the current study illustrates an association between muscle strength, proprioception, and postural stability. These findings suggest considering these potential changes in muscle strength, proprioception, biomechanics, and postural stability in KOA prevention and rehabilitation.

## Data Availability

The original contribution presented in the study are included in the article/Supplementary Material, further inquiries can be directed to the corresponding authors.
