# Supplementary material for: Asymmetries and relationships between muscle strength, proprioception, biomechanics, and postural stability in patients with unilateral knee osteoarthritis
Source: Front Bioeng Biotechnol. 2022 Sep 16;10:922832. doi: 10.3389/fbioe.2022.922832 (PMC9523444; doi:10.3389/fbioe.2022.922832)
Supplement: Supplementary file 1 [file DataSheet1.docx]

**Supplementary Appendix**

**Table 1.** Baseline condition of subjects **(n = 100)**

| **Items** | **PG (n = 50)** | **CG (n = 50)** | ***P* - values** |
| --- | --- | --- | --- |
| Age /age range (years) | 60.88 ± 5.73/ (50-70) | 61.28 ± 5.73/ (50-70) | 0.735 |
| Gender (male/female) | 19/31 | 20/30 | 1.000 |
| Height (m) | 1.61 ± 0.41 | 1.62 ± 0.48 | 0.229 |
| Weight (Kg) | 63.30 ± 4.27 | 62.36 ± 5.72 | 0.583 |
| BMI (Kg/m^2^) | 24.21 ± 1.71 | 23.49 ± 2.02 | 0.057 |
| Duration of disease (months) | 38.12 ± 35.23 | - | - |
| Pain location (left/right) | 25/25 | - | - |
| VAS scores | 3.62 ± 1.49 | - | - |
| K/L grade | 2.32 ± 0.74 | - | - |

Values are mean ± standard deviation or n; BMI, body mass index; K/L, Kellgren/Lawrence; PG, patient group; CG, control group.

**Table 2.** Comparison of parameters between the two groups **(n = 100)**

| Parameters | **PG (n = 50)** | | | **CG (n = 50)** | | |
| --- | --- | --- | --- | --- | --- | --- |
|  | Symptomatic side | Asymptomatic side | Combined | Left side | Right side | Combined |
| Muscle strength (Nm/kg) | 0.92 ± 0.03^#♦^ | 1.03 ± 0.04^♦^ | 0.97±0.03^♦^ | 1.15 ± 0.04^*^ | 1.15 ± 0.05 | 1.15 ± 0.04 |
| Proprioception (°) | 5.72 ± 2.17^#♦^ | 4.86 ± 1.96^♥^ | 5.29±2.03^♦^ | 4.06 ± 1.63^*^ | 4.04 ± 1.57 | 4.05 ± 1.45 |
| FTA (°) | 178.52 ± 5.25^#^ | 176.14 ± 4.20 | 177.33±4.65 | - | - | - |
| FCTP (°) | 3.22 ± 1.34^#^ | 2.64 ± 1.12 | 2.93±1.19 | - | - | - |
| ATE (%) | 32.68 ± 11.62^#♦^ | 27.84 ± 11.56^♥^ | 30.26±11.49^♦^ | 23.28 ± 9.15^*^ | 23.06 ± 9.17 | 23.17 ± 9.09 |
| COP sways areas (mm^2^) | 683.08 ± 470.72^♦^ | | | 348.90 ± 208.31 | | |

Values are mean ± standard deviation; FTA, femorotibial angle; FCTP, femoral condylar–tibial plateau angle; ATE, average trajectory error; COP, the center of pressure; PG, patient group; CG, control group.

Compared to contralateral, ^#^*p* < 0.01; Compared to contralateral, ^*^*p* > 0.05; Compared to CG, ^♦^*p* < 0.01; Compared to CG, ^♥^*p* < 0.05.

**Table 3.** Statistical analysis results of correlation test

| **Parameters** |  | **Sides** | **ATE** | | **COP sways areas** | |
| --- | --- | --- | --- | --- | --- | --- |
|  |  |  | ***r*** | ***p-*value** | ***r*** | ***p-value*** |
| Muscle strength | PG | Symptomatic side | -0.380 | 0.006^#^ | -0.338 | 0.016^*^ |
|  |  | Asymptomatic side | -0.312 | 0.028^*^ | -0.287 | 0.044^*^ |
|  |  | Combined | -0.377 | 0.007^#^ | -0.348 | 0.013^*^ |
|  | CG | Left side | -0.381 | 0.006^#^ | -0.316 | 0.025^*^ |
|  |  | Right side | -0.424 | 0.002^#^ | -0.345 | 0.014^*^ |
|  |  | Combined | -0.405 | 0.004^#^ | -0.343 | 0.015^*^ |
| Proprioception  (degree error) | PG | Symptomatic side | 0.330 | 0.019^*^ | 0.297 | 0.036^*^ |
|  |  | Asymptomatic side | 0.339 | 0.016^*^ | 0.328 | 0.020^*^ |
|  |  | Combined | 0.372 | 0.008^#^ | 0.324 | 0.022^*^ |
|  | CG | Left side | 0.305 | 0.031^*^ | 0.367 | 0.009^#^ |
|  |  | Right side | 0.385 | 0.006^#^ | 0.337 | 0.017^*^ |
|  |  | Combined | 0.356 | 0.011^*^ | 0.358 | 0.011^*^ |
| FTA | PG | Symptomatic side | 0.250 | 0.080 | 0.225 | 0.115 |
|  |  | Asymptomatic side | 0.133 | 0.359 | 0.147 | 0.307 |
|  |  | Combined | 0.199 | 0.166 | 0.183 | 0.204 |
| FCTP | PG | Symptomatic side | 0.133 | 0.356 | 0.150 | 0.298 |
|  |  | Asymptomatic side | 0.099 | 0.494 | 0.130 | 0.367 |
|  |  | Combined | 0.118 | 0.413 | 0.155 | 0.281 |
| VAS | PG | - | 0.302 | 0.033^*^ | 0.318 | 0.025^*^ |

^#^Indicates *p*-value < 0.01; *Indicates *p*-value < 0.05. VAS, visual analogue scale; FTA, femorotibial angle; FCTP, femoral condylar–tibial plateau angle; ATE, average trajectory error; COP, the center of pressure; PG, patient group; CG, control group.
